# Tagless LysoIP for immunoaffinity enrichment of native lysosomes from clinical samples

**DOI:** 10.1172/JCI183592

**Published:** 2024-12-26

**Authors:** Daniel Saarela, Pawel Lis, Sara Gomes, Raja S. Nirujogi, Wentao Dong, Eshaan Rawat, Sophie Glendinning, Karolina Zeneviciute, Enrico Bagnoli, Rotimi Fasimoye, Cindy Lin, Kwamina Nyame, Fanni A. Boros, Friederike Zunke, Frederic Lamoliatte, Sadik Elshani, Matthew Jaconelli, Judith J.M. Jans, Margriet A. Huisman, Christian Posern, Lena M. Westermann, Angela Schulz, Peter M. van Hasselt, Dario R. Alessi, Monther Abu-Remaileh, Esther M. Sammler

**Affiliations:** 1 Medical Research Council (MRC) Protein Phosphorylation and Ubiquitylation Unit, School of Life Sciences, University of Dundee, Dundee, United Kingdom.; 2 Aligning Science Across Parkinson’s (ASAP) Collaborative Research Network, Chevy Chase, Maryland, USA.; 3 Department of Chemical Engineering,; 4 Department of Genetics, and; 5 The Institute for Chemistry, Engineering and Medicine for Human Health (Sarafan ChEM-H), Stanford University, Stanford, California, USA.; 6 Department of Molecular Neurology, University Hospital Erlangen, Friedrich-Alexander-University Erlangen-Nürnberg (FAU), Erlangen, Germany.; 7 Department of Metabolic diseases, Wilhelmina Children’s Hospital, University Medical Center Utrecht, University Utrecht, Utrecht, Netherlands.; 8 Department of Pediatrics, University Medical Center Hamburg-Eppendorf, Hamburg, Germany.; 9 German Center for Child and Adolescent Health, partner site Hamburg, Hamburg, Germany; 10 The Phil & Penny Knight Initiative for Brain Resilience at the Wu Tsai Neurosciences Institute, Stanford University, Stanford, California, USA.; 11 Division of Neuroscience, School of Medicine, University of Dundee, Dundee, United Kingdom.

**Keywords:** Cell biology, Neuroscience, Genetic diseases, Lysosomes, Neurodegeneration

## Abstract

Lysosomes are implicated in a wide spectrum of human diseases, including monogenic lysosomal storage disorders (LSDs), age-associated neurodegeneration, and cancer. Profiling lysosomal content using tag-based lysosomal immunoprecipitation (LysoTagIP) in cell and animal models has substantially moved the field forward, but studying lysosomal dysfunction in patients remains challenging. Here, we report the development of the ‘tagless LysoIP’ method, designed to enable the rapid enrichment of lysosomes, via immunoprecipitation, using the endogenous integral lysosomal membrane protein TMEM192, directly from clinical samples and human cell lines (e.g., induced pluripotent stem cell–derived neurons). Isolated lysosomes were intact and suitable for subsequent multimodal omics analyses. To validate our approach, we applied the tagless LysoIP to enrich lysosomes from peripheral blood mononuclear cells derived from fresh blood of healthy donors and patients with CLN3 disease, an autosomal recessive neurodegenerative LSD. Metabolic profiling of isolated lysosomes revealed massive accumulation of glycerophosphodiesters (GPDs) in patients’ lysosomes. Interestingly, a patient with a milder phenotype and genotype displayed lower accumulation of lysosomal GPDs, consistent with their potential role as disease biomarkers. Altogether, the tagless LysoIP provides a framework to study native lysosomes from patient samples, identify disease biomarkers, and discover human-relevant disease mechanisms.

## Abstract 2

We describe and validate the tagless LysoIP approach to enable the isolation and study of native lysosomes from human peripheral blood mononuclear cells for analysis of biomarkers and human-relevant disease mechanisms.

## Graphical abstract

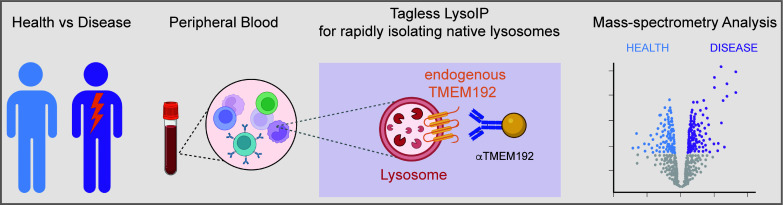

## Introduction

Lysosomes are membrane-bound organelles whose function is critical to maintain cellular homeostasis through nutrient recycling, clearance of cellular waste, and signaling (1–3). Dysfunctional lysosomes are implicated in a wide range of human diseases, in particular, lysosomal storage disorders (LSDs) and neurodegeneration (3–5). Lysosomes make up only 1%–3% of a cell’s volume (6), which makes it challenging to interrogate changes in their molecular content under disease conditions. Recently, the LysoTagIP approach has enabled rapid immunoprecipitation of intact lysosomes, allowing subsequent analyses of their content and leading to a better understanding of human disease (7–10). This approach is based on exogenous expression of the lysosome-resident transmembrane TMEM192 protein containing 3 tandem HA epitopes at its C-terminus (LysoTag) in target cells and mouse models, allowing for rapid lysosome immunoprecipitation (8, 10).

Tagging patient lysosomes is impossible, thus, for studying lysosomal biology and dysfunction in clinical samples, we developed the “tagless LysoIP” method, which enables the enrichment of lysosomes from a wide range of human-derived cells and clinical samples.

The tagless LysoIP approach exploits an antibody capable of recognizing endogenous TMEM192, thereby bypassing the need to exogenously express a LysoTag. Using proteomic, metabolomic, and lipidomic mass spectrometry as well as flow cytometry and enzyme assays, we demonstrate that this method efficiently enriches lysosomes from human cell lines, human peripheral blood mononuclear cells, and human iPSC-derived neurons.

To further validate the utility of our method to study human diseases, we applied the tagless LysoIP to profile the metabolite content of CLN3 patients’ lysosomes. CLN3 disease is the most common form of neuronal ceroid lipofuscinoses (NCLs), a family of neurodegenerative LSDs with childhood onset and progressive degeneration of the brain, including retina and intracellular accumulation of autofluorescent lipopigment (lipofuscin) (11). Recent work using LysoTag mice and cells revealed that the CLN3 protein is required for the clearance of glycerophosphodiesters (GPDs) from lysosomes and that GPDs accumulate in CLN3 knockout mice and cells harboring the LysoTag (8, 9). Here, we show significant accumulation of GPDs in lysosomes derived from patients’ PBMCs using the tagless LysoIP method compared with those from healthy controls; a finding that would have gone unnoticed at the whole cell lysate level. Our results indicate that the tagless LysoIP method is a powerful and rapid approach to enrich lysosomes from clinical samples and other human cells such as iPSC-derived neurons for both biomarker discovery and understanding disease pathology.

## Results

### Development of the tagless LysoIP method.

**Figure 1:**
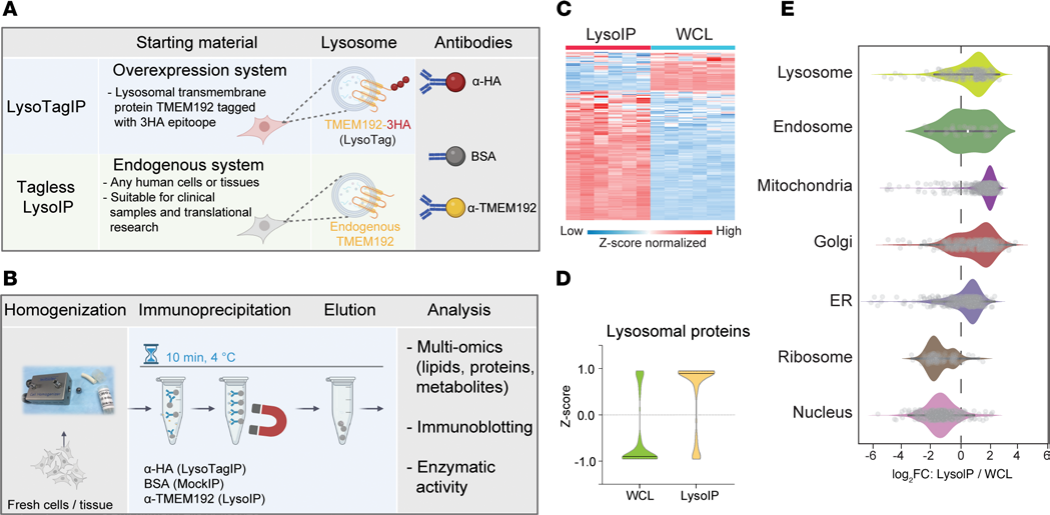
Tagless LysoIP for enriching lysosomes from clinical samples. (**A**) Concept of the tagless LysoIP method via immunoprecipitation of the lysosomal transmembrane TMEM192 protein compared with the LysoTag system, which relies on overexpression of 3HA epitopes at the TMEM192 C-terminus. (**B**) Tagless LysoIP workflow. (**C**) Protein profile heatmap and (**D**) Violin plots of lysosomal proteins enriched via the tagless LysoIP in the immunoprecipitates and whole cell lysates from WT HEK293 cells. (**E**) Organelle profiling demonstrates marked enrichment of lysosomal but also nonlysosomal proteins.

TMEM192 is a 271-residue lysosomal protein, consisting of 4 transmembrane domains, with its N- and C-termini facing the cytoplasm (Supplemental Figure 1A; supplemental material available online with this article; https://doi.org/10.1172/JCI183592DS1). We identified 2 rabbit monoclonal antibodies, TMEM192^AB1^ (Abcam ab186737) and TMEM192^AB2^ (Abcam ab185545) that successfully immunoprecipitate the human TMEM192-3HA protein and detect its presence with immunoblotting in lysates of HEK293 cells stably expressing the protein (Supplemental Figure 1, B and C). Epitope analysis revealed that TMEM192^AB1^ recognizes C-terminal residues from 235 to 250, while TMEM192^AB2^ recognizes those between 200 and 235 (Supplemental Figure 1C). To test for the ability of the identified antibodies to enrich for lysosomes from detergent-free homogenized cell lysates, we covalently coupled them to magnetic beads and incubated each conjugated antibody with homogenates from WT HEK293 cells and HEK293 expressing the LysoTag (TMEM192-3HA). As controls, we used magnetic beads coupled to HA antibodies (HA-IP) as well as BSA (MockIP) (Figure 1A). Cells were homogenized using a ball-bearing homogenizer in an isotonic potassium phosphate-buffered saline (KPBS) with protease and phosphatase inhibitors (Figure 1B). To minimize leakage, the homogenization, immunoprecipitation, and wash steps were optimized to take approximately 10 minutes as in our original LysoTagIP protocol and apart from using different magnetic bead–coupled antibodies or BSA alone, there was no difference in the overall procedure (Figure 1B) (10).

To rigorously assess the enrichment of lysosomal proteins in immunoprecipitates, whole cell lysates (WCLs) and IPs were initially analyzed using high-resolution data-independent acquisition (DIA) liquid chromatography/tandem mass spectrometry (LC-MS/MS). TMEM192^AB1^ and TMEM192^AB2^ IPs clustered closely together in both WT and LysoTag-expressing cells (Supplemental Figure 2A). As expected, the HA-IP from LysoTag cells yielded robust enrichment of lysosomal proteins (Supplemental Figure 2B). TMEM192^AB1^ also markedly enriched lysosomes in WT and LysoTag cells, albeit to a lower extent than observed in HA-IPs from LysoTag cells (Supplemental Figure 2B). TMEM192^AB2^ antibody was less efficient at enriching lysosomes than TMEM192^AB1^ antibody (Supplemental Figure 2, B and C). Thus, all subsequent work was performed using TMEM192^AB1^ antibody, and the approach was termed the “tagless LysoIP method”.

Further proteome analyses established that lysosome-annotated proteins are enriched in TMEM192^AB1^ IP from WT HEK293 cells (Figure 1, C–E and Supplemental Figure 2, B and D). These include key lysosomal proteins such as LAMP1, LAMTOR1, TMEM55B, CTSC, CTSD, and GBA1 (Supplemental Figure 2E).

Despite the clear enrichment of lysosomal content, organelle profiling revealed the presence of nonlysosomal proteins in the tagless LysoIP, especially from endosomal, mitochondrial, and Golgi compartments (Figure 1E). This was further illustrated in the volcano plot analysis (Supplemental Figure 2D) and when looking at the relative intensities of representative cytoplasmic (Tubulin) and other organelle proteins (VDAC1; mitochondria, ACBD3; Golgi) in LysoIP compared with MockIP and WCL samples (Supplemental Figure 2E). Altogether, these data suggest that the tagless LysoIP enriches for lysosomes while minor fractions from other compartments are also present.

To test that the presence of mitochondrial proteins in the LysoIPs was not due to nonspecific binding of the TMEM192^AB1^ antibody to mitochondria, we costained WT HEK293 cells with the TMEM192^AB1^ antibody and either a lysosomal (LAMP1) or mitochondrial marker (ATPB) (Supplemental Figure 3, A and B). This showed that binding of the antibody is lysosome specific, suggesting that the presence of mitochondrial proteins in the LysoIP is likely due to other factors, including contact sites between mitochondria and lysosomes than mere binding of the TMEM192^AB1^ antibody to mitochondria. We also performed costaining experiments with an endoplasmic reticulum marker (Calnexin) and TMEM192 as well as a Golgi marker (GM130) and TMEM192 (Supplemental Figure 3, C and D). While the staining of the Calnexin and TMEM192 antibodies was largely distinct, some colocalization was observed. GM130 did not overlap with TMEM192.

To confirm that the enriched lysosomes are intact, we preincubated HEK293 cells with the pH-dependent lysosomal fluorescent dye LysoTracker prior to cell homogenization (Supplemental Figure 3E). IPs were subsequently analyzed by flow cytometry, which demonstrated a marked increase of LysoTracker-labeled lysosomes in LysoIPs compared with MockIPs, indicating small molecule retention during the tagless LysoIP process (Supplemental Figure 3F). 

Pretreatment of HEK293 cells with bafilomycin to suppress the acidification of lysosomes reduced the LysoTracker signal by approximately 50%. Moreover, the activity of cathepsin D, a luminal lysosomal enzyme, was substantially higher in LysoIP compared with MockIP and WCL, further indicating that the tagless LysoIP enriches intact lysosomes (Supplemental Figure 3G).

**Figure 2:**
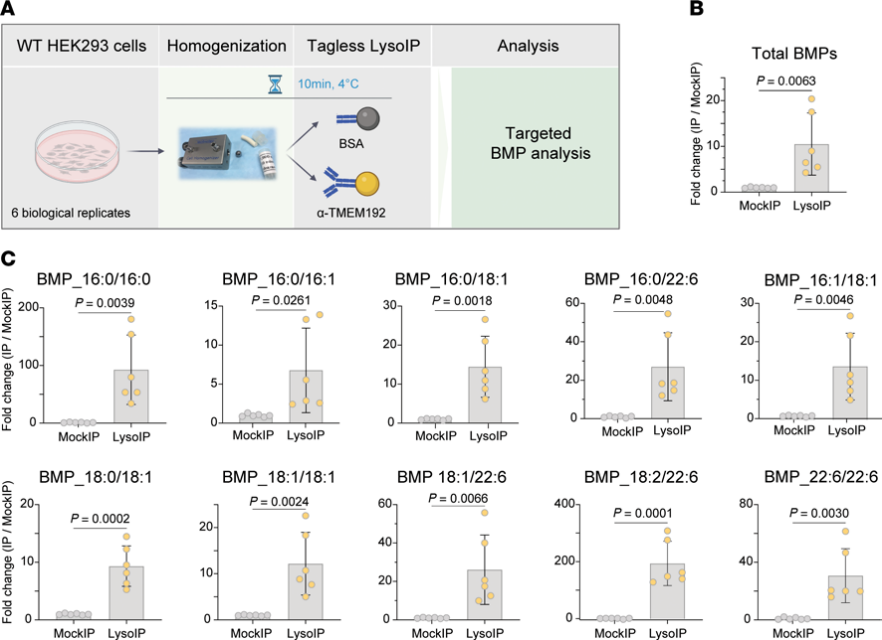
Bis(monoacylglycero)phosphate lipids are enriched in LysoIP samples from WT HEK293 cells. (**A**) Experimental design to study lysosomal lipids using tagless LysoIP followed by targeted Bis(monoacylglycero)phosphate **(**BMP) analysis. (**B**) Targeted analysis of accumulated BMPs in lysosomes derived from WT HEK293 cells using tagless LysoIP (*n* = 6). Relative enrichment of total BMPs quantified in the LysoIP samples and the MockIP samples. Data presented as mean ± SD (*n* = 6). Statistical analysis was performed using 2-tailed unpaired *t* test. (**C**) Enrichment of specific BMP species in the LysoIP samples.

We next performed targeted lipidomic analysis for negatively charged lysosomal phospholipid bis(monoacylglycero)phosphate (BMP) species, as these are relevant biomarkers for a range of neurodegenerative conditions (Figure 2A). For example, BMPs are elevated in urine of patients with LSDs (12) and may have utility as a urine biomarker in leucine-rich repeat kinase 2 associated (LRRK2-associated) Parkinson’s disease (PD) (13–15). We were able to robustly measure BMPs in HEK293 cells and found that they were enriched in LysoIP compared with MockIP samples (Figure 2, B and C).

**Figure 3:**
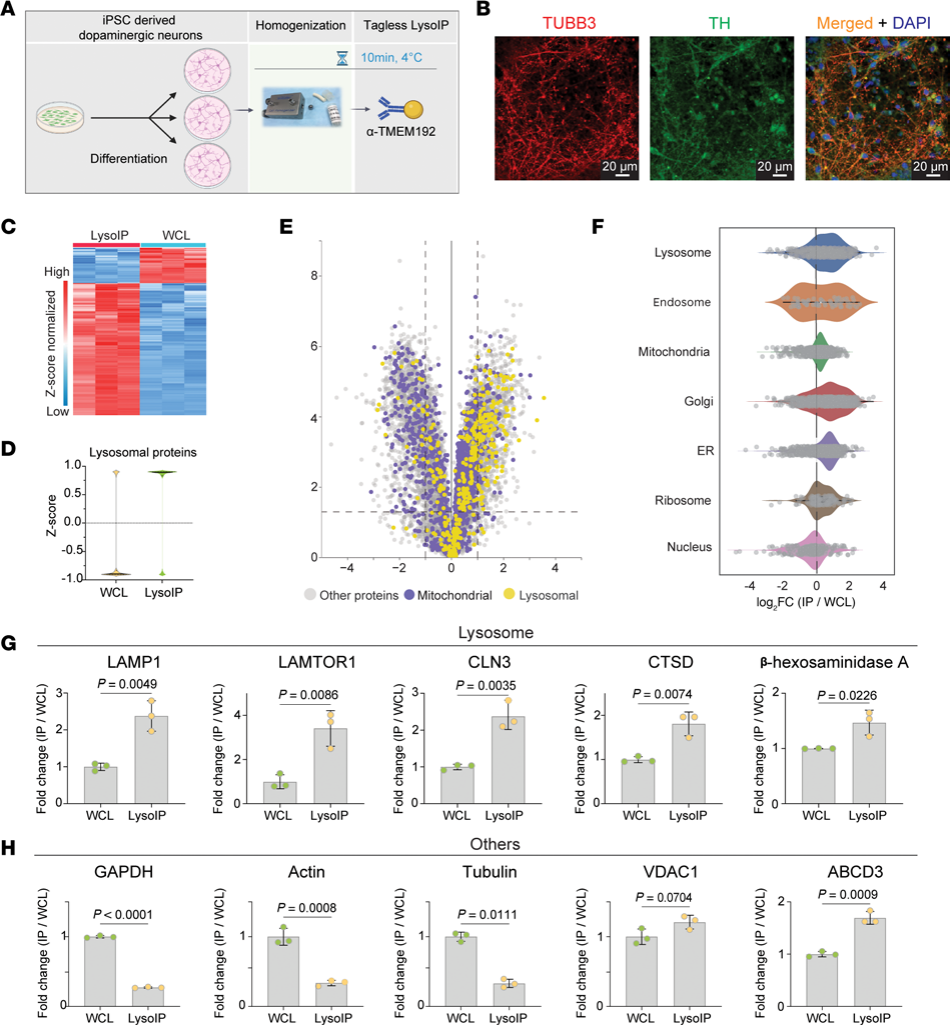
Tagless LysoIP from human iPSC-derived dopaminergic neurons. (**A**) Workflow. (**B**) 78-day–old iPSC-derived dopaminergic neurons were stained for tyrosine hydroxylase ([TH] green), a marker of dopaminergic neurons, TUBB3 (red), a neuronal-specific βIII tubulin marker, and DAPI (blue), indicating nuclear DNA. (**C**) Protein profile heatmap. (**D**) Violin plots of lysosomal proteins enriched via the tagless LysoIP in the immunoprecipitates from iPSC-derived dopaminergic neurons. (**E**) Volcano plot of proteins enriched/depleted in the LysoIPs compared with whole cell extracts. Data were analyzed via Curtain: https://curtain.proteo.info/#/cefde280-9d48-4157-b959-cf2c3b0cc9e7 (**F**) Organelle profiling demonstrates enrichment of lysosomal proteins and modest enrichment of other organelles. Bar plots of representative (**G**) lysosomal proteins and (**H**) cytosolic, mitochondrial, and Golgi proteins in LysoIPs and their respective whole cell extracts. Data presented as mean ± SD (*n* = 3). Statistical analysis was performed using 2-tailed unpaired *t* test.

To test the tagless LysoIP method in relevant cellular models of human disease, we performed proteome analysis on lysosomes enriched from iPSC-derived dopaminergic neurons that have previously been characterized (16, 17) (Figure 3, A and B). Heatmap clustering and Z-score analysis revealed clear enrichment of lysosomal proteins in the LysoIPs compared with WCLs (Figure 3, C and D). This was also seen in the volcano plot analysis comparing LysoIP with whole-cell proteomic content (Figure 3E), which again highlighted the presence of mitochondrial proteins in the LysoIPs. As with the HEK293 experiments, proteins from other organelles were also present, although to a lesser extent than the lysosomal ones (Figure 3F); the lysosomal proteins LAMP1, LAMTOR1, CLN3, CTSD, and β-hexosaminidase A were enriched in LysoIPs compared with WCLs (Figure 3G). Cytoplasmic markers, including GAPDH, actin, and tubulin were depleted, while the Golgi-associated ABCD3 protein was enriched in LysoIPs (Figure 3H). The mitochondrial VDAC1 protein was not enriched in LysoIPs (Figure 3H).

### The tagless LysoIP method enriches lysosomes from PBMCs.

**Figure 4:**
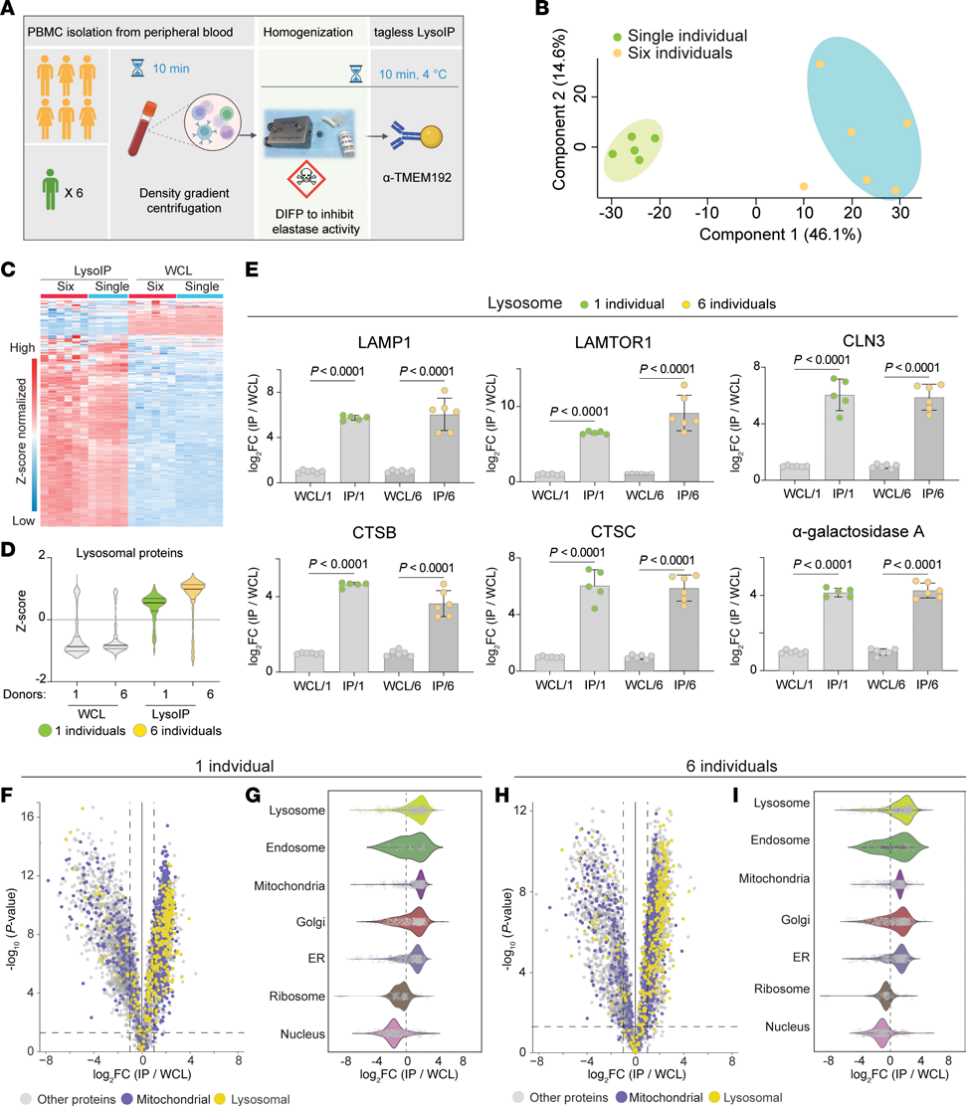
Tagless LysoIP in human peripheral blood mononuclear cells. (**A**) Workflow (**B**) Principal component analysis of DIA mass spectrometry data of tagless LysoIPs from PBMCs from a single donor (6 replicates) and 6 different donors. (**C**) Protein profile heatmap. (**D**) Lysosomal protein enrichment. (**E**) Bar plots of representative lysosomal transmembrane (top panel) and intraluminal (lower panel) proteins in LysoIPs from 1 and 6 donors compared with the respective whole cell extracts. Data presented as mean ± SD (*n* = 6). Multiple unpaired *t* tests with 2-stage step-up method of Benjamini, Krieger, and Yekutieli (1% FDR) used to correct for multiple comparisons between the groups. Volcano and violin plots of LysoIPs compared with whole cell extracts and organelle profiling from the single (**F** and **G**) and multiple (**H** and **I**) donor experiments. Data were analyzed via Curtain: https://curtain.proteo.info/#/b573afb8-df5d-4a39-b5ba-88eb9488820d (**F**), https://curtain.proteo.info/#/83006f89-901d-44db-b078-5b8504844ee6 (**H**).

Human peripheral blood mononuclear cells (PBMCs) are routinely isolated from human peripheral blood for clinical and biomarker studies. These comprise a heterogeneous group of white blood cells including lymphocytes (T cells, B cells, and NK cells), monocytes, and dendritic cells (18). We explored the feasibility of performing lysosomal enrichment from PBMCs isolated from fresh whole blood via the tagless LysoIP method, as depicted in Figure 4A. We first undertook pilot experiments with and without the potent elastase protease inhibitor, diisopropylfluorophosphate (DIFP) in the cell homogenization buffer, as PBMCs are often contaminated with trace amounts of neutrophils that contain high levels of elastases that can degrade proteins in extracts (19) (Supplemental Figure 4A). The addition of DIFP in the homogenization buffer markedly increased the detectability of key organelle marker proteins (LAMP1, TMEM55B, LAMTOR1, GM130, and HSP60), not only in the IPs but also in WCLs (Supplemental Figure 4B). Cytoplasmic proteins such as tubulin and GAPDH, on the other hand, were significantly depleted in LysoIPs compared with WCLs (Supplemental Figure 4B). This emphasizes the importance of maintaining DIFP in the homogenization buffer for all PBMC tagless LysoIP experiments. It should be noted that DIFP is a highly toxic organophosphate and thus should be handled with appropriate care, in a fume hood, and according to site-specific standard operating procedures.

To test technical reproducibility and interindividual variability of the tagless LysoIP methodology in PBMCs, we performed 6 tagless LysoIPs from a single healthy donor (technical replicates) as well as tagless LysoIPs from 6 healthy individuals (biological replicates) (Figure 4 and Supplemental Figure 5). The samples were analyzed in parallel by DIA LC/MS-MS, as described in the methods section in ‘sample preparation and analysis for quantitative proteomics’. On average, over 5,000 unique proteins were identified in the LysoIP samples, except for 1 replicate from the single donor experiment (1,003 identified proteins) that was subsequently excluded from further analysis (data not shown). Principal component analysis revealed that replicates from the single and multiple donor tagless LysoIP experiments clustered closely together (Supplemental Figure 5A), but, as expected, there was more variation between LysoIPs from multiple donors in the biological replicate experiment compared with the technical replicate experiment from a single donor (Figure 4B). MockIPs were only performed for the technical variability single donor experiment, but also clustered closely but distinctly from the LysoIPs and WCLs (Supplemental Figure 5A). Heatmap clustering of the DIA-MS data revealed that levels of lysosomal proteins were similar in all LysoIP samples (Figure 4C). Likewise, violin plots (Z-score normalized) confirmed strong enrichment of lysosomal proteins in the tagless LysoIP experiments from human PBMCs compared with WCLs (Figure 4D). Volcano plot analysis confirmed enrichment of lysosomal proteins in the LysoIP for the technical replicates (single donor) (Figure 4F) and biological replicate (multiple donors) experiments (Figure 4H) compared with corresponding WCLs.

Levels of luminal (CTSB, CTSD, and GBA1) and transmembrane (LAMP1, LAMTOR1, and CLN3) lysosomal proteins displayed 4-to-10–fold enrichment in LysoIPs compared with whole cell extracts (Figure 4E). We noted that Golgi (ACBD3 & GM130) and mitochondrial (HSP60,VDAC1) markers were also enriched 4-to-6–fold in PBMC LysoIPs compared with WCLs (Supplemental Figure 5, B and C). Cytosolic markers such as Tubulin and Actin were depleted in PBMC LysoIP samples (Supplemental Figure 5D). This may reflect the capture of the newly synthesized, tagless LysoIP antigen TMEM192 as it traverses the secretory pathway. Organelle profiling of PBMC LysoIPs for both single (Figure 4G) and multiple donors (Figure 4I) showed similar results to what had been observed in HEK293 cells (Figure 1E) with regard to the presence of mitochondria and other organelles.

**Figure 5:**
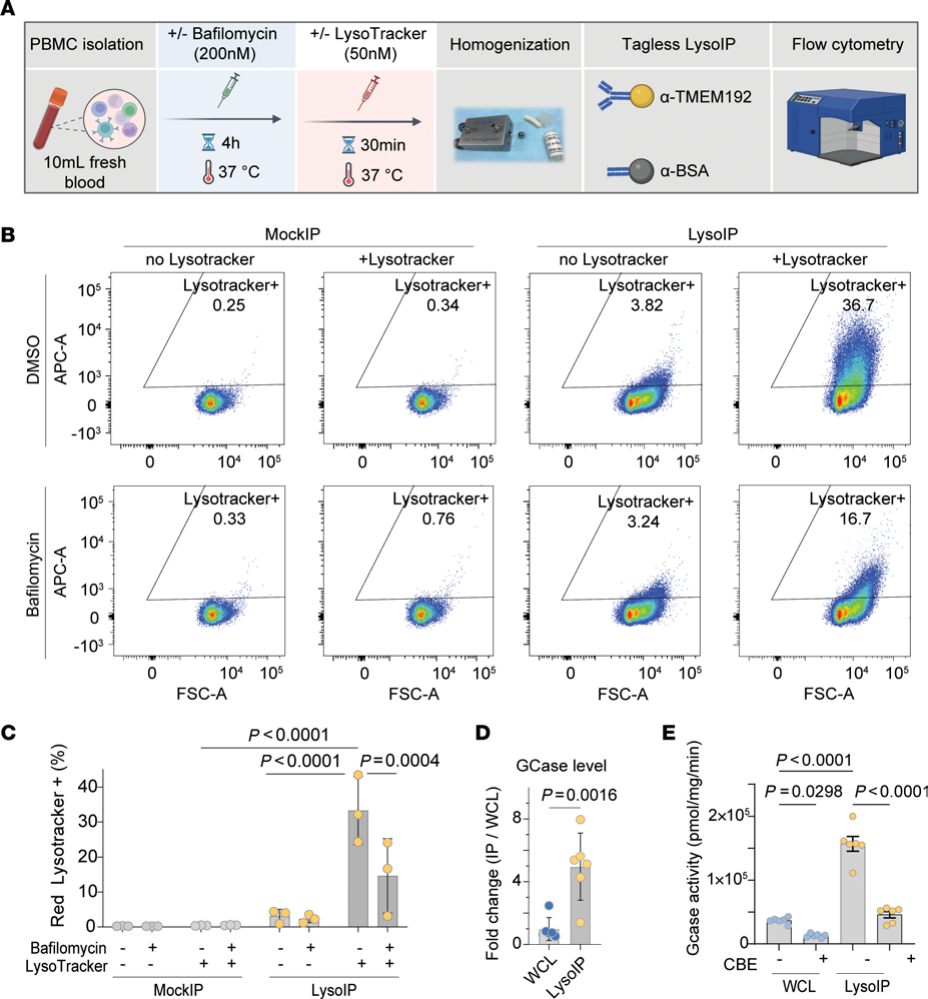
Tagless LysoIP enriches intact and functional lysosomes from healthy donor PBMCs. (**A**) Workflow of flow cytometry analysis of magnetic beads bound to lysosomes enriched via tagless LysoIPs or MockIPs from PBMC homogenates pretreated with and without the V-ATPase inhibitor bafilomycin A1 (200 nM), prior to staining with Red Lysotracker (50 nM). (**B**) Representative scatter plot from 1 of the 3 donors, (Y-axis) and bead size (Forward scatter, FSC, X-axis), (**C**) Quantification of the percentage of beads positive for Lysotracker fluorescence. Data presented as mean ± SD (*n* = 3 donors) and analysed by ordinary 2-way ANOVA with Dunnet’s multiple comparison test. (**D**) GCase (glucocerebrosidase, GBA1) protein enrichment quantified by DIA mass-spectrometry in LysoIPs from 6 donors. Data presented as mean ± SD (*n* = 6). 2-tailed unpaired *t* test. (**E**) GCase activity measured by 4-methylumbelliferone (4-MU) assay in lysosomes enriched from PBMCs from 6 donors. Data presented as mean ± SD and 2-way ANOVA with Dunnet’s test for multiple comparison.

To confirm that the enriched lysosomes are intact, we preincubated PBMCs with the pH-dependent lysosomal fluorescent dye LysoTracker prior to cell homogenization and tagless LysoIP and subsequently analyzed the LysoIPs by flow cytometry (Figure 5A). Consistent with isolation of intact lysosomes in the HEK293 cells (Supplemental Figure 3), the tagless LysoIP enriched lysosomes from PBMCs that retained the LysoTracker signal, which was responsive to bafilomycin treatment (Figure 5, B and C). Furthermore, protein levels (Figure 5D) and catalytic activity (Figure 5E) of the intraluminal lysosomal hydrolase GCase (glucocerebrosidase, GBA1) were enriched in LysoIPs compared with WCL fractions. Of note, homozygous GBA1 variants cause Gaucher’s disease, a common LSD, while heterozygous GBA1 variant carrier status increases risk for PD (20).

### The application of tagless LysoIP in clinical settings.

Encouraged by the ability of our method to enrich intact lysosomes from healthy donor PBMCs for multimodal profiling, we explored the feasibility and utility of the tagless LysoIP methodology to identify clinically relevant lysosomal biomarkers in LSDs.

We turned to CLN3 disease, a devastating early onset neurodegenerative LSD, as we had previously shown a causal link between the lack of CLN3 and lysosomal GPD accumulation in human cell lines and animal models (8, 9). In total, fresh peripheral blood samples were collected from 10 individuals, 5 patients with CLN3-associated NCL disease and 5 sex-matched controls. Four of the patients carried a common 1 kb deletion mutation in the homozygous state and presented with typical juvenile onset with retinal disease and additional complex neurodegenerative symptoms. A single patient possessed compound heterozygous mutations that may represent a partial loss of function and presented with adult onset (3rd decade) retinal-only disease (Table 1). Due to ethical and practical considerations, our controls were mostly young healthy adults and not aged-matched children.

**Figure 6:**
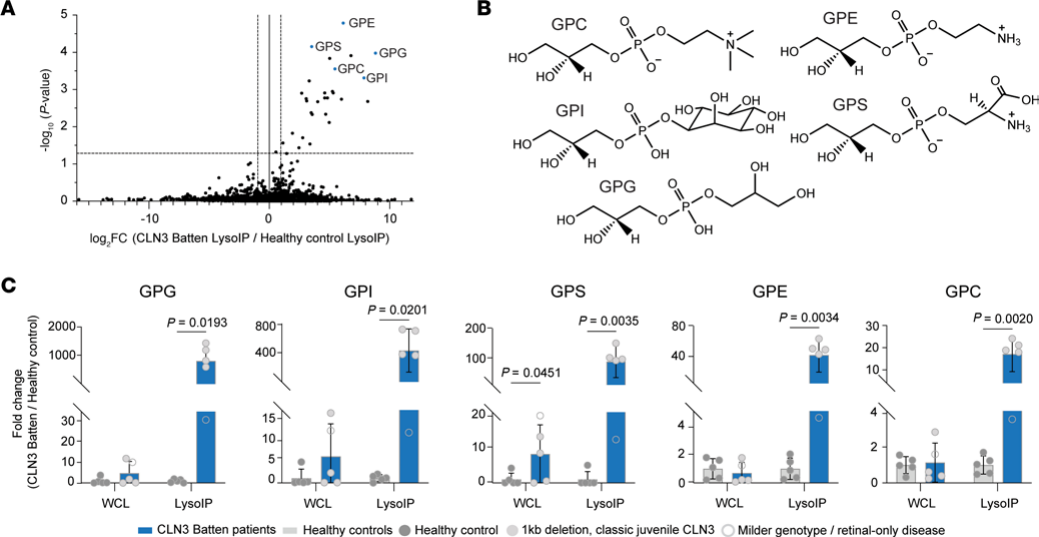
Striking GPD accumulation in lysosomes enriched from patients with CLN3 disease. (**A**) Volcano plot comparing untargeted metabolomic LysoIP data derived from PBMCs from patients with CLN3 and healthy controls. (**B**) The chemical structure of the annotated GPDs in this study. (**C**) Targeted analyses of GPDs in the LysoIPs and corresponding WCLs. Data presented as mean ± SD (*n* = 5). Multiple unpaired *t* tests with 2-stage step-up method of Benjamini, Krieger, and Yekutieli (1% FDR) used to correct for multiple comparisons between the groups.

We first undertook untargeted metabolomic mass spectrometry to profile the metabolite content of enriched lysosomes, as described previously (8) (Figure 6A). This revealed marked elevation of 5 GPDs in patients’ lysosomes that we were able to unambiguously annotate, namely glycerophosphoglycerol (GPG), glycerophosphoinositol (GPI), glycerophosphocholine (GPC), glycerophosphoethanolamine (GPE), and glycerophosphospingosine (GPS) (Figure 6, A and B). Targeted analyses in both LysoIPs and corresponding whole-cell fractions demonstrated massive accumulation of these metabolites in CLN3-disease lysosomes, with an average of 17-to-830–fold accumulation of all 5 annotated GPDs (GPG 830-fold, GPI 440-fold, GPS 95-fold, GPE 42-fold, GPC 17-fold) in the lysosomes from patients with CLN3 who had the classic 1 kb deletion compared with healthy controls (Figure 6C). At the whole-cell level, a significant increase was only observed for 1 GPD metabolite, namely, GPS (8-fold), whereas the remaining GPDs (GPG, GPI, GPC, and GPE) did not show a significant increase despite showing a potential trend in some cases. These data are consistent with our findings in cell culture and mouse models (8, 9). Interestingly, the patient with the milder disease associated with compound heterozygous mutations in CLN3 still displayed an enrichment of GPDs in the LysoIP compared with its corresponding WCL fraction. However, this enrichment was to a lower extent than what was observed in patients with the classical, more severe genotype and disease manifestation, but still higher when compared with healthy controls (4-to-30–fold) (Figure 6C). These results indicate that the tagless LysoIP can potentially be used to identify and monitor disease biomarkers in human LSDs.

## Discussion

Lysosomal dysfunction has been implicated in a myriad of human diseases. While tools to profile lysosomal content have been established for cell culture and animal models by expressing a tagged lysosomal membrane protein for organelle immunoprecipitation (7–10), profiling lysosomal content from patients has remained a great challenge.

Here, we report the development of the “tagless LysoIP’’ method that allows the rapid enrichment of lysosomes via immunoprecipitation using an antibody against the integral lysosomal membrane protein TMEM192. After validating the method and confirming the intactness of the enriched lysosomes, a requirement to preserve their content, we applied the tagless LysoIP for the multimodal analyses of lysosomes from human cells including cultured cell lines, iPSC-derived neurons, and human PBMCs. To test the utility of the method in uncovering molecular pathways involved in disease pathology at the subcellular level, we isolated and profiled lysosomes from PBMCs collected from patients with CLN3 disease and healthy individuals. In striking agreement with our cell culture and mouse studies, we identified a massive accumulation of GPDs in the lysosomes of patients with CLN3 (8, 9). Of importance, the elevation of GPDs was barely observed and mostly not significant (except for GPS) at the whole-cell level. These robust results emphasize the need of lysosomal enrichment via the tagless LysoIP method to study diseases where lysosomes are implicated, including LSDs and neurodegenerative conditions, as most of the disease-relevant alterations could otherwise be missed.

Interestingly, one of the patients with CLN3 included in this study had a much-delayed age of onset with 2 compound heterozygous mutations in *CLN3* causing an atypical mild phenotype restricted to retinal pathology only (Table 1). This individual still had elevated GPD metabolites within the lysosome, but at least 5-fold (between 4.8-to-43–fold) lower than in patients with CLN3 that are homozygous for the common 1 kb deletion that results in a complete loss of function. This example illustrates the quantitative nature of the tagless LysoIP and its potential to measure lysosome-restricted biomarkers and correlate them to disease phenotypes, especially in cases with atypical mutations (21, 22).

While the tagless LysoIP represents a major step toward applying subcellular profiling to clinical samples, the LysoTag approach that relies on overexpressing the 3HA-tagged TMEM192 remains the default option for other applications, as it allows for the isolation of more content with higher purity (Supplemental Figure 2B).

While our data indicate that the TMEM192^AB1^ antibody is relatively specific for the lysosome (Supplemental Figure 3, A–D) and enables enriching intact lysosomes (Figure 5 and Supplemental Figure 3), proteins from other organelles are coenriched in the LysoIPs (Figures 1, 3, and 4). This could be due to some degree of nonspecific binding but also because of contact sites between organelles (23, 24).

Our unpublished observations were that using cryopreserved PBMCs was less efficient in enriching lysosomes compared with using freshly isolated PBMCs. However, this should be explored further in conjunction with optimized PBMC cryopreservation and reconstitution methods, as the ability to use cryopreserved material for the tagless LysoIP could substantially widen its applicability, e.g., where fresh samples are not available.

Moreover, the TMEM192^AB1^ antibody is specific for the human protein and does not recognize the mouse TMEM192 protein due to amino acid sequence differences in the epitope. Thus, the current version of the tagless LysoIP is unsuitable for experiments in mouse models. Future work should aim at screening more antibodies that can resolve both issues and expand the utility of our optimized platform. Alternatively, a conditional LysoTag mouse model could be deployed (8).

Altogether, our data show that the tagless LysoIP approach is a robust methodology for enriching lysosomes from clinical samples and human cell lines, including human iPSC-derived neurons, which provides a framework for studying lysosomal dysfunction in human diseases. The potential use of this technology spans from biomarker discovery and drug screening to uncovering the function of lysosomal gene products. The presence of proteins from other organelles would, however, require extensive validation when it comes to decoding the lysosomal content.

To determine the ultimate use as a biomarker to assess efficacy of experimental therapies, cross-sectional and longitudinal analyses of samples from a larger cohort of patients with CLN3 disease with age and sex matched controls are mandatory. In addition, these patients should be well characterized by established clinical scoring systems, e.g., Unified Batten Disease Rating Scale (25) or Hamburg CLN3 ophthalmic rating scale (26). We would also suggest expanding the LysoIP mass spectrometry analysis of clinical samples from metabolites such as GPDs to proteins and lipids, including targeted BMP measurements. Given that lysosomal dysfunction is a hallmark of cognitive decline in PD and other neurodegenerative disorders (27), the tagless LysoIP represents an important asset for translational research for studying lysosomes using clinical and iPSC-derived cell lines across a wide range of human diseases.

## Methods

### Sex as a biological variable.

Sex was not considered as a biological variable due to the overall small number of human participants, which was too small for grouping by sex. Also, while participants of both sexes are represented in the clinical part of the study (Table 1), the selection and enrolment of participants was mainly based on feasibility and availability of patients with CLN3 disease, as the condition is very rare.

### Generation of stable LysoTag cell lines.

Detailed protocol for preparing the stable cell lines used in this study are described at ref. 28 (steps 1–22). Briefly: HEK293 cells (The American Type Culture Collection. Catalog no. CRL-1573, RRID:CVCL 0045) were transfected with lentiviral constructs expressing either TMEM192-3×HA (LysoTag) (https://www.addgene.org/102930/) or a control vector expressing only 3×HA (MockTag) (DU70022) and cultured in DMEM containing 10% (by volume) FBS supplemented with L-glutamine and penicillin and streptomycin. After 24 hours, cells were subjected to selection with puromycin (2 μg/mL) for approximately 72 hours.

### LysoTagIP method for HEK293 cells.

We have slightly adapted the previously described LysoTagIP method (10) with the main difference being employing an Isobiotec cell breaker for the homogenization step. Detailed protocol for this slightly modified method is described at ref. 28 (Steps 23–52). Briefly: cells stably expressing LysoTag or MockTag (RRID:CVCL_D6SR and RRID:CVCL_C8A7) were cultured in the continued presence of puromycin (2 μg/mL) in 15 cm diameter dishes to 90% confluency. Cells were scraped into Potassium Phosphate Buffer Saline (KPBS) containing 1× Complete Protease Inhibitor cocktail (Catalog no. 11836170001) and 1× PhosSTOP phosphatase inhibitor cocktail (Catalog no. 4906845001). An aliquot (5% by volume) of the cell suspension was removed and cells pelleted at 1,500*g* and frozen in liquid nitrogen and stored at –80 °C for subsequent proteomic, lipidomic, or metabolomic analysis. This fraction is termed the “whole cell extract”. The remaining cell suspension was homogenized using an Isobiotec cell breaker. Homogenates were cleared of debris by centrifugation at 1,500*g* for 2 minutes at 4°C. The remainder of the supernatant containing cellular organelles was collected and transferred to a clean 1.5 mL Eppendorf tube containing 100 μL of 50% (by volume) slurry of anti-HA magnetic bead (Thermo Fisher Catalog no. 88837). The mixture was incubated at 4 °C for 5 minutes on an orbiter ensuring constant gentle agitation. After incubation, the tube containing the mixture was placed on a magnet for 30 seconds and the supernatant was removed. The beads were washed 3 times with ice cold KPBS, aliquoted, and snap frozen in liquid nitrogen and stored in –80 °C until further processing (see Method sections ‘Sample preparation and analysis for quantitative proteomics’, and ‘Sample preparation and analysis for quantitative lipidomics’).

### Generation of monoclonal TMEM192 antibody–coupled magnetic beads.

Detailed protocol for generating monoclonal TMEM192 antibody-coupled magnetic beads is described at ref. 29. For each coupling reaction, 20 mg of MyOne Epoxy Dynabeads (Invitrogen, 34001D) was conjugated with 600 μg of rabbit monoclonal TMEM192 antibody (TMEM192^AB1^ [Abcam ab186737] and/or TMEM192AB2 [Abcam ab185545]). The antibodies were purchased in PBS and their concentrations adjusted to 1.2 mg/mL. A total of 20 mg of magnetic beads were resuspended in 1 mL of sterile Milli-Q water, vortexed, and sonicated in a water bath sonicator for 5 minutes, after which the Milli-Q water was removed. Beads were resuspended in 1 mL of sterile Milli-Q water and the sonication was repeated. After sonication, the Milli-Q water was removed and the beads were mixed with 0.5 mL of 1.2 mg/mL antibody solution (total amount 600 μg of the antibody) and vortexed, after which 500 μL of buffer C2 (3 M Ammonium Sulphate ((NH4)2SO4) in 0.1 M Sodium Phosphate buffer pH 7.4) was added. The bead suspension was vortexed again and then was incubated on a Thermo mixer at 37°C, shaking at 1,500 rpm for 20 hours. After coupling, beads were washed once with buffer HB (100 mM Glycine pH 11.3, 0.01% Tween-20), followed by 1 wash with buffer LB (200 mM Glycine pH 2.8, 0.01% Tween-20), and 3 washes with buffer SB (50 mM Tris-HCl (NH2C(CH2OH)3·HCl) pH 7.4 with 140 mM NaCl and 0.1% Tween-20). Beads were then resuspended in 2 mL of the storage buffer (50 mM Tris-HCl (NH2C(CH2OH)3·HCl) pH 7.4 with 140 mM NaCl, 0.1% Tween-20 and 0.2% NaN3) to a final concentration of 10 mg/mL and stored at 4°C. We used the beads stored in this manner for up to 1 month.

### Generation of BSA-coupled magnetic beads for MockIP controls.

The method is identical to the method above describing generation of monoclonal TMEM192 antibody-coupled magnetic beads, except that 600 μg of BSA buffer exchanged into PBS was coupled to 20 mg of MyOne Epoxy Dynabeads (Invitrogen, 34001D).

### Tagless LysoIP method for HEK293 cells and iPSC-derived dopaminergic neurons.

The method is identical to the LysoTagIP method described above, except that monoclonal TMEM192 antibody-coupled magnetic beads and BSA-coupled magnetic beads (MockIP) replace the HA-magnetic beads with the same amount and volume of extract and magnetic beads.

The isogenic controls (WT) for the iPSC-derived A53T-α-synuclein dopaminergic neurons (16, 17) were differentiated following the Kriks protocol (30) and as described in recent publications (17, 31). The minimum iPSC quality control panel data is available in Zenodo (https://doi.org/10.5281/zenodo.14827155). At an age of 84 days after start of the differentiation, cells were harvested and processed for tagless LysoIP as described above.

### Tagless LysoIP method for human PBMCs.

Detailed protocols for isolation of PBMCs and tagless LysoIP are described with embedded video method at ref. 32. Briefly, fresh blood (5–60 mL) was collected from participants into K2EDTA vacutainers and PBMCs were isolated using density gradient centrifugation as described previously (19) and in protocols.io (33). Isolated cells were briefly washed 3 times with PBS and cells resuspended in 0.8 mL KPBS containing 1X Complete Protease Inhibitor cocktail (Catalog no. 11836170001) and 1X PhosSTOP phosphatase inhibitor cocktail (Catalog no.4906845001) (volume of resuspension buffer independent of volume of starting blood volume) and supplemented with freshly diluted 0.5 mM diisopropylfluorophosphate made up from 0.5 M stock in isopropanol to inhibit elastase proteases derived from low levels of contaminating neutrophils. Note that diisopropylfluorophosphate is highly toxic and must be handled in a fume hood and waste disposed in 2% (w/v) NaOH. An aliquot (5% by volume) of the cell suspension was removed and cells were pelleted at 1500*g* and frozen in liquid nitrogen and stored at –80 °C for subsequent proteomic, lipidomic, or metabolomic analysis. This fraction is termed the “whole cell extract”. The remaining resuspended PBMCs were homogenized using an Isobiotec cell-breaker. The remaining steps for the immunoprecipitation are identical to those described above for the tagless LysoIP method.

### Study participants and blood sample collection.

Between 10 and 60 mL of peripheral blood was collected from healthy volunteers for setting up the method. From November 2022 and February 2024, between 8 and 10 mL of fresh peripheral blood was collected from a total of 5 participants with CLN3 Batten disease: 2 were recruited from the Department of Metabolic Diseases, Wilhelmina Children’s Hospital, University Medical Center Utrecht, Utrecht University, Utrecht in the Netherlands, and 3 from the Department of Pediatrics, University Medical Center Hamburg-Eppendorf, Hamburg, Germany. Additionally, a total of 5 sex-matched young healthy donors were recruited from both clinical sites as controls (Table 1).

Diagnosis was confirmed upon identification of biallelic homozygous pathogenic variants in the CLN3 gene (CLN3; Chr16(GRCh37)c.461-280_677+382del966). In the case of the patient with adult onset (5th decade) retinal-only disease, the diagnosis was based on the combination of biallelic compound heterozygous variants in CLN3 (c.391_392del; p. (Ser131fs) and CLN3 (c.969G>A; r. (spl?)), clinical symptoms with characteristic optical coherence tomography (OCT) abnormalities (34, 35) and characteristic fingerprint abnormalities on the electron microscopy of the skin (36). Demographics including sex, age at disease onset, clinical phenotype, and CLN3 genotype were collected (Table 1). Please see paragraph below in ‘Study approval’ for more details regarding IRB approvals and informed consent procedures that were all in keeping with the Declaration of Helsinki principles.

### Flow cytometry assay.

Detailed protocol for the flow cytometry assay is described in ref. 37. HEK293 cells (15 cm dish, 90% confluent) and PBMCs (isolated from 15 mL of serum and divided into 4 equal aliquots) were treated ± 200 nM Bafilomycin at 37°C for 3.5 hours and cells were then incubated for a further 30 minutes ± 50 nM Deep Red LysoTracker at 37°C. Both bafilomycin and LysoTracker were dissolved in DMSO at a 1000 × concentration, and, for the minus-bafilomycin and/or minus-Lysotracker conditions, the equivalent volume of DMSO was added. Tagless LysoIP were performed as described above. The isolated lysosomes bound to the magnetics beads were diluted 1 in 10 in KPBS and transferred to FACS tubes and analyzed on a BD LSR Fortessa cytometer at 647 nm excitation measuring the emission at 668 nm, the optimal wavelength for Deep Red LysoTracker.

### Cathepsin D activity assay.

Detailed protocol for the Cathepsin D assay is described in ref. 38. WT HEK293 cells (15 cm dish, 90% confluent) were subjected to tagless LysoIP as described above. The isolated lysosomes bound to the magnetics beads were diluted 1 in 10 in KPBS. Protein levels in the whole cell extracts and LysoIP were determined using the ultrasensitive Bicinchoninic acid assay (BCA) method (39). A total of 2 μg of protein from the whole cell extract and the LysoIP, MockIP, and WCL were aliquoted into wells in a 96-well plate and assayed using a fluorometric Cathepsin D activity assay kit (Abcam. Catalog no. Ab65302) that is based on quantifying the hydrolysis of the preferred cathepsin-D substrate sequence GKPILFFRLK(Dnp)-D-R-NH2) labeled with MCA fluorescent dye. Cathepsin D substrate hydrolysis released fluorophore fluorescence was measured on a Clariostar Plate Reader (Ex/Em= 328 nm/460 nm).

### Glucocerebrosidase-1 enzyme activity assay.

Detailed protocol for the glucocerebrosidase-1 enzyme activity assay is described in ref. 40. Glucocerebrosidase-1 (GCase) enzyme activity was measured by monitoring the cleavage of the fluorescent substrate 4methylumbelliferyl-β-D-glucopyranoside (4-MUG). Detailed protocol for the GCase enzyme activity assay is described in ref. 41. Briefly, PBMCs were isolated from 15 mL fresh peripheral blood from 6 healthy volunteers and subjected to tagless LysoIP as described above. The isolated lysosomes and the whole cell pellets were suspended in 1% (v/v) Triton X-100 lysis buffer, cleared by centrifugation, and protein concentrations were determined using the ultrasensitive BCA method (39). A total of 5 μg of protein from the whole cell extract and 1 μg of protein from the LysoIPs were aliquoted into a 96-well plate in duplicates, and incubated with 500 μM 4-MUG, in assay buffer (0.15 M citrate-phosphate buffer, pH 5.4, 0.25% (w/v) sodium taurocholate, 1 mM EDTA, 0.5% (w/v) BSA), in the presence or absence of 300 μM conduritol β-epoxide (CBE, glucocerebrosidase-1 inhibitor), at 37°C. After a 1 hour incubation, the reaction was stopped by addition of 1 M glycine, pH 12.5, calibrators (0–10 μM 4methylumbelliferone [4-MU]) were aliquoted into empty wells of the plate, and the fluorescence intensity was measured on a Pherastar plate reader (Ex/Em = 350/460 nm). GCase activity was estimated from the generated calibration curve as the amount of released fluorophore (4-MU) per mg of protein extract per minute of reaction.

### Immunofluorescence assay.

Detailed protocol for the immunofluorescence assay is described in ref. 42. Briefly, HEK293 cells (ATCC. Catalog no. CRL1573, RRID:CVCL_0045) were grown on Poly L-lysine coated 22 × 22 glass coverslips in 6-well 3.5 cm diameter plates. For fixation, medium was aspirated and cells were fixed in 3 mL of 4% (w/v) paraformaldehyde in PBS for 10 minutes at room temperature. Fixed cells were then washed 3 times at 5 minute intervals with 0.2% (w/v) BSA dissolved in PBS. Cells were permeabilized with 1% (v/v) Nonidet P40 diluted in PBS for 10 minutes at room temperature. Permeabilized cells were blocked with 1% (w/v) BSA in PBS for 1 hour, then incubated with primary antibody at 1:1,000 dilution in PBS for 1 hour at room temperature in a dark chamber. The combination of the primary antibodies used is Mouse anti-LAMP1 (Santa Cruz, Catalog no. sc-20011. RRID:AB_626853) and Rabbit anti-TMEM192 (Abcam, Catalog no. Ab186737. RRID:AB_3095637) or Mouse anti-ATPB (Abcam, Catalog no. Ab14730. RRID:AB_301438) and Rabbit anti-TMEM192 (Abcam, Catalog no. Ab186737. RRID:AB_3095637) or Mouse anti-Calnexin (Santa Cruz, Catalog no. sc-46669. RRID:AB_626784) and Rabbit anti-TMEM192 (Abcam, Catalog no. Ab186737. RRID:AB_3095637) or Mouse anti-GM130 (BD Biosciences, Catalog no. 610822. RRID:AB_398141) and anti-TMEM192 (Abcam, Catalog no. Ab186737. RRID:AB_3095637) or anti-TH (Sigma-Aldrich, Catalog no. AB5986, RRID:AB_92190) and anti-TUBB3 (BioLegend, Catalog no. 801202, RRID:AB_2313773). This is followed by 3 washes with 0.2% (w/v) BSA at 5 minute intervals. Cells were then incubated for 1 hour in a dark chamber with a mixture of secondary antibodies containing Alexa Fluor 488 donkey anti-mouse (Invitrogen, Catalog no.A21206. RRID:AB_2535792) and Alexa Fluor 594 goat anti-rabbit (Invitrogen, Catalog no. A11012. RRID:AB_2534079) at 1:500 dilution and Hoechst 33342 (Thermo Fisher Scientific, Catalog no. 62249) at 1:1,000 in PBS. Cells were washed again 3 times with 0.2% (w/v) BSA, rinsed in MilliQ water, and mounted on glass microscope slides with VECTASHIELD antifade mounting media (Vector Laboratories, H1000). Slides were then imaged using Leica TCS SP8 MP Multiphoton Microscope using a 40× oil immersion lens choosing the optimal imaging resolution with 1-pixel size of 63.3 nm × 63.3 nm.

### SDS-PAGE and immunoblotting.

Protein concentrations were determined using BCA assay and diluted in NuPAGE LDS sample buffer (4×) and further supplemented with 10% 2-mecraptoethanol (v/v). Between 2 and 30 μg of sample and a protein molecular ladder were loaded onto a commercial NuPAGE Bis-Tris 4%–12% gel. Gels were run in 1× MOPS buffer at 90V for 20 minutes and then further at 130V until dye-front reached the bottom of the gels. For immunoblot analysis the gels were placed on a 0.45 μm between 2 nylon sponges and filter papers soaked in transfer buffer. Protein transfer was carried out over 90 minutes at 90V at 4°C. Transferred membranes were incubated with Ponceau S stain to evaluate transfer success and to aid in cutting the membranes. Ponceau was washed away with TBST (50 mM Tris-HCl pH 7.5, 150 mM NaCl, 0.1% (v/v) Tween-20) and the membrane was blocked with 5% milk in TBST (w/v) for 1 hour. The membranes were then washed 3 × 15 minutes in TBST under agitation. The membranes were then incubated under agitation with the primary antibody in 5% BSA in TBST (w/v, 0.02% sodium azide) overnight at 4°C. Primary antibodies used were LAMP1 (RRID:AB_626853), TMEM55B (RRID:AB_2879391), LAMTOR1 (RRID:AB_10860252), GM130 (RRID:AB_2797933), α-tubulin (RRID:AB_1904178), GAPDH (RRID:AB_627679), HSP60 (RRID:AB_2295614), and HA (RRID:AB_390918) as stated in Supplemental Figures 1 and 4. The next day, the membranes were washed with washed 3 × 15 minutes in TBST under agitation. The membranes were then incubated in the secondary antibodies (LI-COR) at 1:20,000 in 5% milk in TBST for 1 hour under agitation. Membranes were washed 3 times in TBST before imaging on LI-COR Odyssey CLx scanner. Acquired images were visualized and quantified on Image Studio Lite (version 5.2.) and analyzed and visualized using GraphPad Prism (version 10).

### Sample preparation and analysis for quantitative proteomics.

Detailed protocol for the processing of the whole cell extract, LysoTagIP, and LysoIP is described in ref. 43. A detailed protocol for the data-independent mass spectrometry analysis is described in ref. 44. The stored and frozen whole cell extract pellets and the LysoTagIP, LysoIP, and MockIP samples from WT and LysoTag HEK293 cells (RRID:CVCL_0045 and RRID:CVCL_D6SR) PBMCs were resuspended in 0.1 mL of 2% (w/v) (sodium dodecyl sulfate) SDS, 20 mM HEPES pH 8.0 containing 1 × Complete Protease Inhibitor cocktail (Catalog no. 11836170001) and 1 × PhosSTOP phosphatase inhibitor cocktail (Catalog no. 4906845001). Tryptic digestion was carried out using S-Trap-assisted Oncolumn tryptic digestion (45, 46). The digested tryptic peptides were then analyzed on Orbitrap Exploris 480, tims-TOF Pro, tims-TOF SCP and Orbitrap Astral mass spectrometers. The details of LC column, DIA isolation window acquisition schemes, and mass spectrometer data acquisition parameters are provided in supplemental file MSSettings. The raw MS data were further processed using the DIA-NN (1.8.1) (47) against the Uniprot Human database (downloaded January 2023; 20381 entries with isoforms) in a library free mode. DIA-NN database search parameters for each dataset are provided in supplemental file MSSettings. The output files from DIA-NN search were further processed using Perseus software suite (version 1.6.15.0) (48) for differential analysis 2-sided *t* test, lysosomal annotated proteins were further Z-score normalized and subsequently used for supervised heatmap clustering and violin plot representation of LysoIP, LysoTag-IP and WCLs. Further, data visualization was performed using CURTAIN tool (49) and GraphPad Prism (version 10).

### Sample preparation and analysis for quantitative lipidomics.

Detailed protocol for the processing of the whole cell extract, LysoTagIP and LysoIP is described in ref. 50. The stored and frozen whole cell extract pellets and the LysoTagIP, LysoIP and MockIP samples were resuspended in 1 mL LC-MS–grade chloroform/methanol (2:1 by volume) supplemented with 1 μg/mL Splashmix internal standard (Avanti, no. 330707-1EA). Lipids were profiled using an Acentis Express C18 150 × 2.1 m column (Sigma-Aldrich, 53825-U) and the ID-X Tribrid mass spectrometer. Unbiased differential analysis was performed by LipidSearch and Compound Discoverer. Normalization was performed by constant median after blank exclusion. Targeted BMP analyses were performed from an adapted protocol (51) using an Agilent C18 column (Agilent Technologies 821725-90) and Ultivo triple quadrupole mass spectrometer.

### Sample preparation and analysis for quantitative metabolomics.

Detailed protocol for the processing of the whole cell extract, LysoTagIP, and LysoIP is described in ref. 52. The stored and frozen whole cell extract pellets and the LysoTagIP, LysoIP, and MockIP samples were resuspended in LC-MS grade 80% methanol (v/v) with isotopically labeled amino acids (Cambridge Isotope Laboratories, Inc. Catalog no. MSK-A2-1.2). Metabolites were profiled by liquid chromatography/mass spectrometry (LC/MS) using a SeQuant ZIC-pHILIC 150 × 2.1 mm column (Sigma-Aldrich 1504600001) and an ID-X Tribrid mass spectrometer.

Unbiased differential analysis was performed in Compound Discoverer (Thermo Fisher Scientific). Rigorous quantification of metabolite abundance was performed by TraceFinder.

### Statistics.

Perseus 1.6.15.0 version was used for proteomics data analysis and CURTAIN tool was used for proteomic data visualization. Python 3.11.5 was used to generate organelle enrichment plots (script: https://zenodo.org/records/11281488). GraphPad Prism (version 10) for macOS and Windows, GraphPad Software, Boston, Massachusetts, USA, www.graphpad.com was used for data visualization and statistics. Statistical test used include 2-tailed unpaired t test. Multiple unpaired *t* tests with 2-stage step-up method of Benjamini, Krieger, and Yekutieli (1% FDR) used to correct for multiple comparisons between the groups. 2-way ANOVA with Dunnet’s test for multiple comparison and 1-way ANOVA with Dunnet’s HSD post hoc was used for multiple comparison analysis between the groups. 1-way ANOVA with Tukey’s HSD post hoc was used for multiple comparison analysis between the groups. A *P* value less than 0.05 was considered significant. Workflow figures and schematics were created using BioRender.com and further edited using Adobe Illustrator 2024.

### Study approval.

The study was approved by the respective local ethics committees: Medical ethics committees of the Ärztekammer Hamburg, Germany (PV7215) and the NedMec, to which the UMC Utrecht is affiliated (METC, 23-268/A). Patients’ or parents’ written informed consent was obtained according to the Declaration of Helsinki (1991). Additional, nonclinical research ethics approval was in place for experiments using blood from healthy volunteers to set up the methodology (University of Dundee, SMED REC Number 22/84), and written informed consent was obtained from participants.

### Data availability.

All data needed to evaluate the conclusions in the paper are present in the manuscript and Supplemental Material. Supporting data values associated with the main article and supplemental material are included in the Supporting Data Values file, with separate tabs for each applicable figure panel. All the primary data presented here have been deposited in publicly accessible repositories. All data files except for proteomic data (raw data files and their quantitation and statistical analysis) have been deposited in Zenodo (https://doi.org/10.5281/zenodo.14827155). Proteomic data have been deposited in the ProteomeXchange PRIDE repository (identifier: PXD052082; https://proteomecentral.proteomexchange.org/cgi/GetDataset?ID=PXD052082). All plasmids and antibodies generated at the MRC PPU at the University of Dundee can be requested through our website (https://mrcppureagents.dundee.ac.uk/). Plasmid request requires a universal material transfer agreement (MTA) that can be completed online at the time of plasmid request. For the purpose of open access, the authors have applied a CC BY 4.0 public copyright license to all Author Accepted Manuscripts arising from this submission. The data, code, protocols, and key lab materials used and generated in this study are listed in a Key Resource Table alongside their persistent identifiers in Zenodo (https://doi.org/10.5281/zenodo.14827155).

## Author contributions

The study was conceived by DRA, MAR and EMS. Methodology was developed and substantially contributed to by DS, PL, S Glendinning, RSN, WD, FL, KZ, ER, RF, EB, S Gomes, FZ, FAB, MJ, MAH, DRA, MAR, and EMS. Data acquisition and analysis was conducted by DS, PL, S Glendinning, RSN, WD, KZ, ER, CL, KN, RF, EB, FZ, FAB, MJ, CP, LMW, and EMS. About 80% of experiments were conducted by DS and analyzed by DS, RSN, WD, ER, and MJ. Data was visualized by DS, PL, S Gomes, RSN, WD, KZ, ER, RF, EB, MJ, and EMS. Figure layout, editing, and harmonization was done by DS and EMS. Funding was acquired by AS, PMVH, DRA, MAR, and EMS. Project administration and supervision were conducted by DRA, MAR, and EMS. The original draft was written by DS, PL, RSN, DRA, MAR, and EMS. Reviewing and editing was done by DS, PL, S Gomes, RSN, WD, KZ, ER, RF, CL, KN, EB, S Glendinning, FZ, FAB, MJ, JJMJ, MAH, CP, LMW, AS, PMVH, DRA, MAR, and EMS. More than 90% of edits on the original draft were done by DS, MAR, DRA, and EMS. The video method for tagless LysoIP was conceived, directed, and produced by DS and SE.

## Supplementary Material

Supplemental data

Unedited blot and gel images

Supporting data values

## Figures and Tables

**Table 1:**
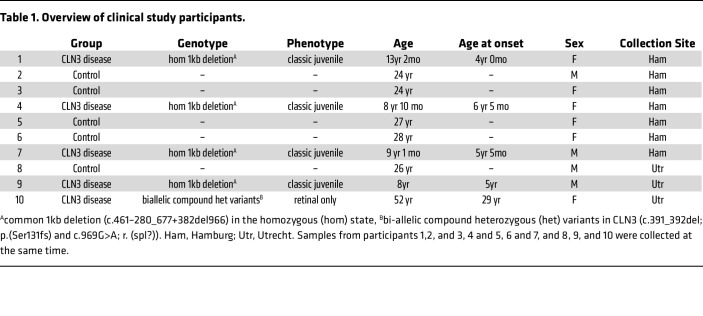
Overview of clinical study participants.

## Version 1 12/26/2024

In-Press Preview

## Version 2 02/17/2025

Electronic publication
