# Supplementary material for: Tagless LysoIP for immunoaffinity enrichment of native lysosomes from clinical samples
Source: J Clin Invest. 2024 Dec 26;135(4):e183592. doi: 10.1172/JCI183592 (PMC11827837; doi:10.1172/JCI183592)
Supplement: Supplemental data [file jci-135-183592-s131.pdf]

Supplementary Figures

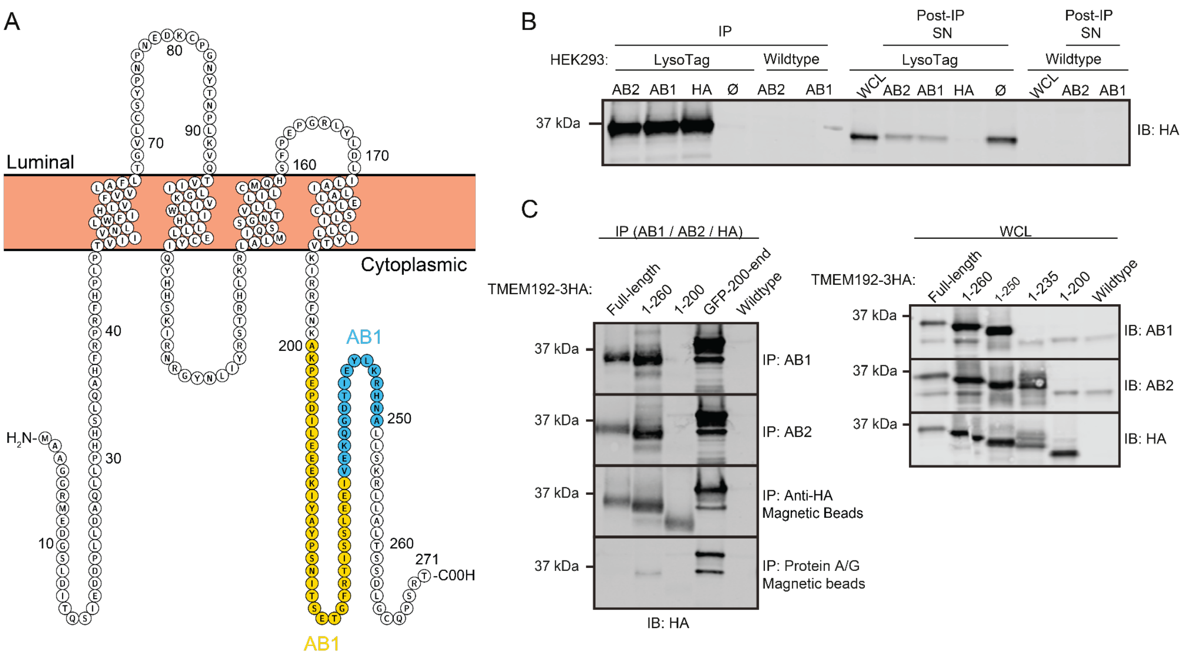

Supplemental Figure 1. Characterization of 2 commercially available TMEM192 antibodies for their ability to immunoprecipitate TMEM192. (A) Domain architecture of TMEM192 protein with regions containing antibody epitopes highlighted in blue (AB1, Abcam ab186737) and yellow (AB2, Abcam ab185545) (Figure created using Protter wlab.ethz.ch/protter). **(B)** Ability of the two TMEM192 antibodies to immunoprecipitate overexpressed TMEM192. TMEM192-3HA was immunoprecipitated from HEK293 lysates using AB1, AB2 and HA beads, using empty beads (Ø) and HEK293 lysates without overexpression (Wildtype) as negative controls. Immunodepletion was additionally assessed in the post-IP supernatants (SN)**(C)** Epitope analysis to map the respective TMEM192 antibody binding sites. Full-length and the indicated C-terminal truncations of TMEM192-3HA were overexpressed and immunoprecipitated using AB1, AB2 and HA beads to confirm the presence of the epitopes within the C-terminal region of TMEM192. Further analysis was performed by immunoblotting of a panel of overexpressed truncations (1-260, 1-250, 1-235, 1-200) and full-length TMEM192-3HA using AB1, AB2 and HA antibodies.

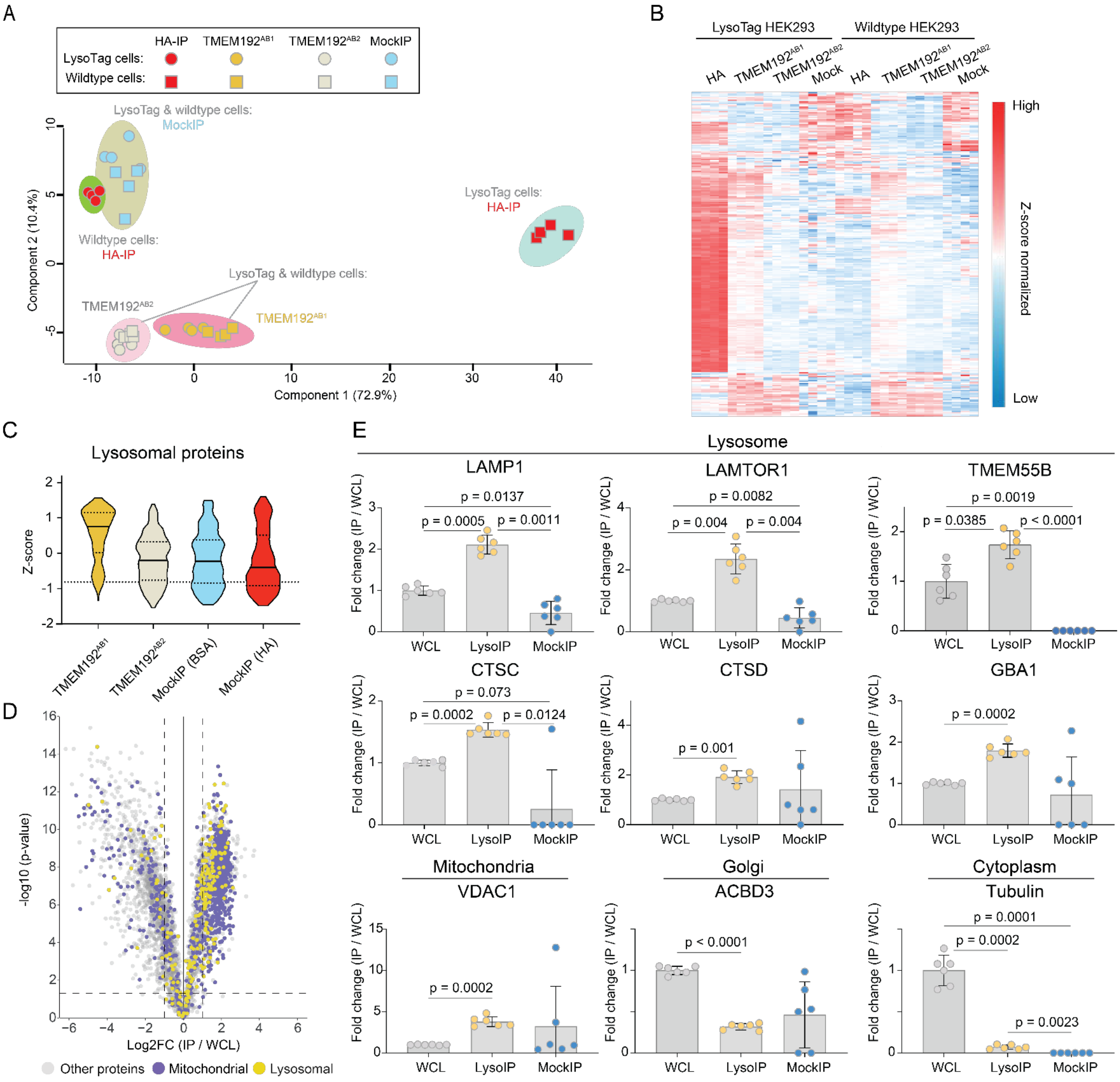

Supplemental Figure 2. TMEM192 antibody selection for tagless LysoIP. (A) Principal component analysis of DIA mass spectrometry data of immunoprecipitates using TMEM192^{AB1}/ TMEM192^{AB2}/ HA-/ BSA-coupled magnetic beads in wildtype HEK293 cells (filled squares n = 4) and LysoTag HEK293 cells (filled circles n = 4). **(B)** Heatmap clustering of lysosomal annotated proteins denoting significant enrichment of lysosomal proteins in HA-IP and TMEM192^{AB1} IP (Z-score normalized). **(C)** Similar to B, the violin plot depicting the enrichment of curated lysosomal-annotated proteins described in Supplemental Dataset 1 using the different coupled magnetic beads in wildtype HEK293 cells (n = 4). **(D)** Volcano plot showing the fold enrichment/depletion of proteins in the LysoIPs (n = 6, p-value adjusted for 1% permutation-based FDR correction, s0 = 0.1). The yellow dots indicate known lysosomal-annotated proteins, and the purple dots indicate mitochondrial-annotated proteins curated from databases described in Supplemental Datasets 1 and 2 (https://doi.org/10.5281/zenodo.11085342). Curtain link: https://curtain.proteo.info/#/f097ad91-ed49-4a29-bb4b-ed8037009a04. **(E)** Bar charts depicting the relative enrichment of indicated protein markers for lysosome, mitochondria, Golgi and cytoplasm. Data presented as mean ± SD (n = 6). One-way ANOVA with Tukey's HSD post-hoc was used for multiple comparison analysis between the groups.

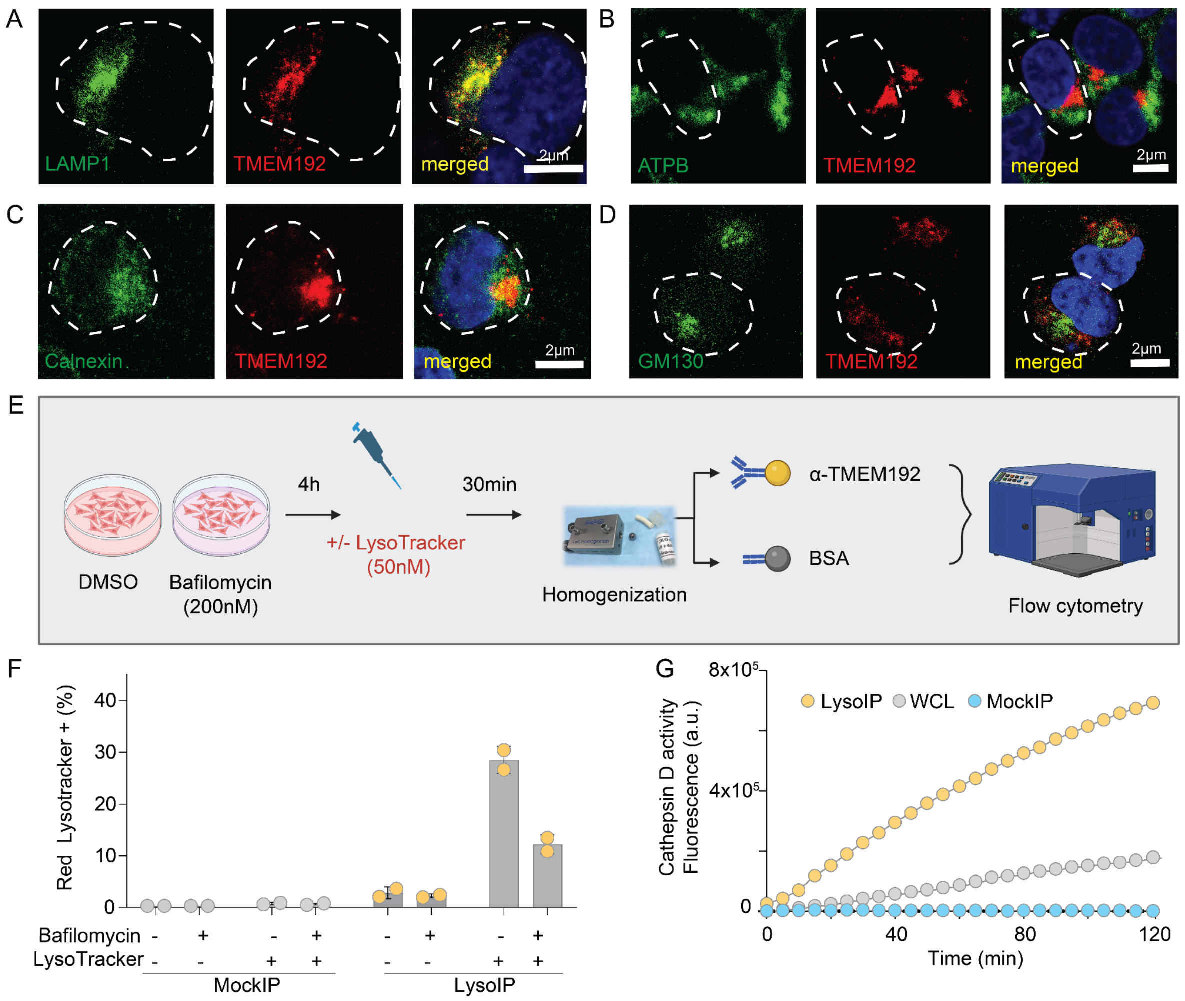

Supplemental Figure 3. Isolation of functional lysosomes using the endogenous TMEM192-IP in HEK293 cells. Co-staining of anti-TMEM192^{AB1} is with a lysosomal **(A)**, mitochondrial **(B)**, ER **(C)** and Golgi marker **(D)** Scale bar = 2 µm. Schematic of workflow for flow cytometry analysis in HEK293 cells. **(F)** Representative scatter plot of the percentage of beads positive for the LysoTracker from one experimental replicate. Data presented as mean ± SD (n = 2). **(G)** Measurement of Cathepsin D activity in LysoIP, MockIP and WCLs of HEK293 cells (n = 2).

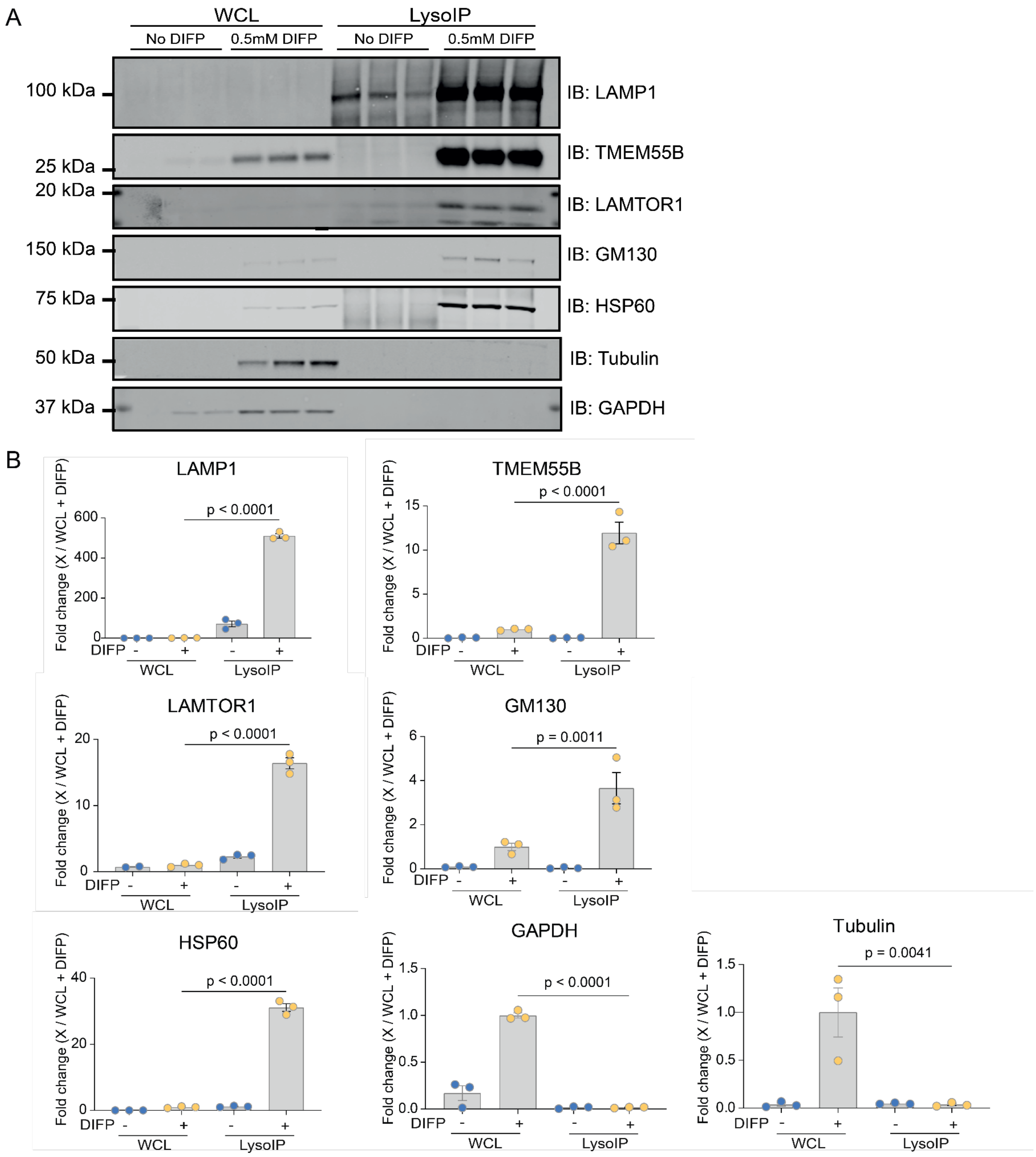

Supplemental Figure 4. Diisopropylfluorophosphate is required to prevent protein degradation in PBMCs (A) PBMCs were treated with 0.5 mM diisopropylfluorophosphate (DIFP) to prevent rapid degradation of proteins. Whole-cell lysates (2 µg) as well as the resuspended IPs (2 µg) were subjected to immunoblotting with the lysosomal (LAMP1, TMEM55B, LAMTOR1), Golgi (GM130), cytosolic (α-tubulin, GAPDH) and mitochondrial (HSP60) markers. The data shown is from 3 healthy male donors. The same samples were run on two gels and a composite image of both gels is shown. **(B)** Quantitative immunoblotting analysis. The graph represents ratios of each condition against DIFP treated WCL (mean ± SEM, n = 3). Two-way ANOVA with Dunnet’s HSD post-hoc was used for multiple comparison analysis between the groups.

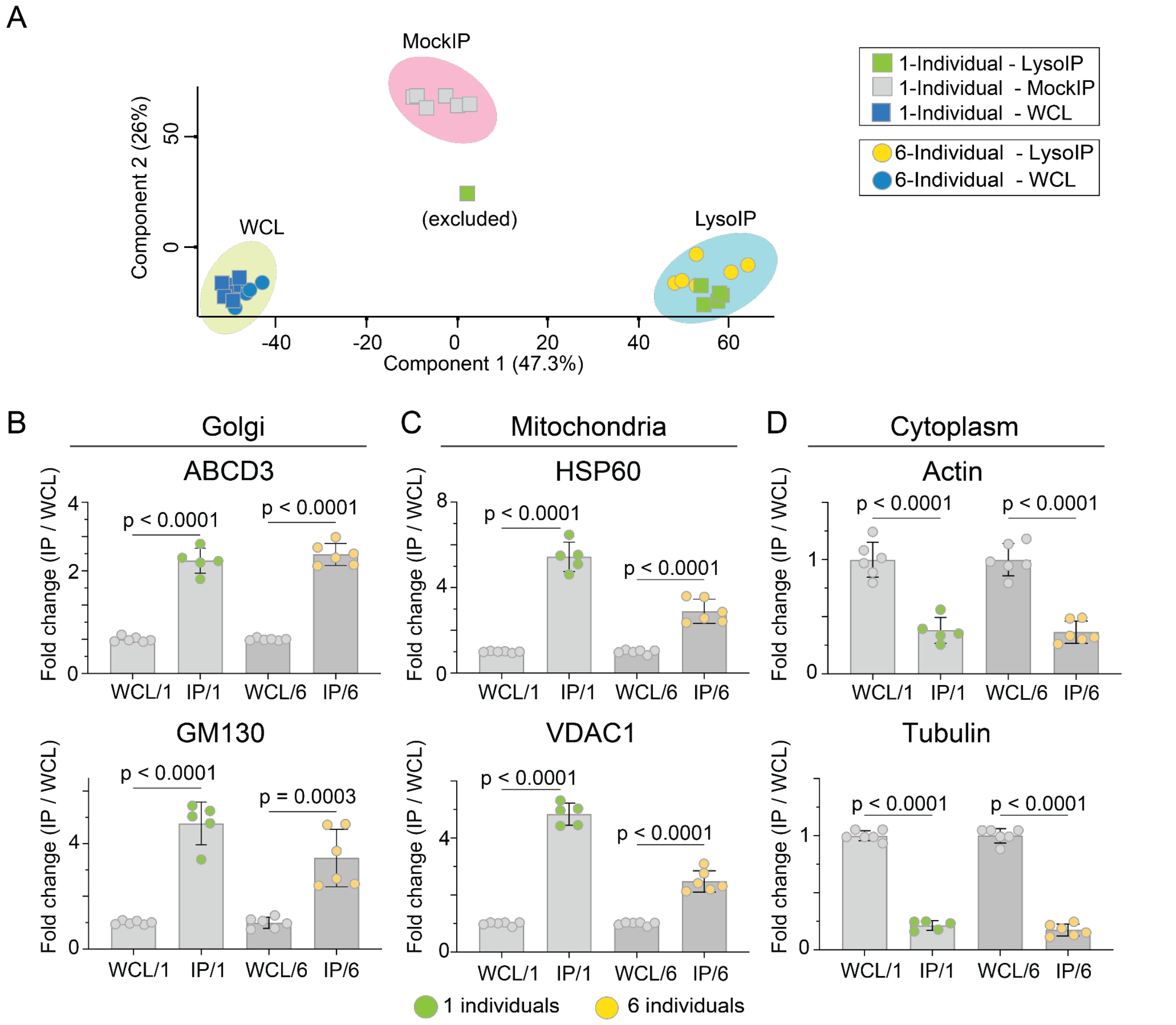

Supplemental Figure 5. Characterization of the tagless LysoIP in PBMCs from healthy donors: (A) Principal component analysis of DIA mass spectrometry data of LysoIP, MockIP (for single donor experiment only) immunoprecipitates as well as WCLs. **(B)** Bar graphs of representative proteins from Golgi-, mitochondria and cytosol enriched/depleted in the LysoIPs. The graph represents ratios of IP/WCL (mean ± SD, n = 6). Data presented as mean ± SD (n = 5 / 6). Multiple unpaired t-tests with two-stage step-up method of Benjamini, Krieger and Yekutieli (1% FDR) used to correct for multiple comparisons between the groups.
