# Supplementary material for: Tagless LysoIP for immunoaffinity enrichment of native lysosomes from clinical samples
Source: J Clin Invest. 2024 Dec 26;135(4):e183592. doi: 10.1172/JCI183592 (PMC11827837; doi:10.1172/JCI183592)

Supplemental Figure 1B

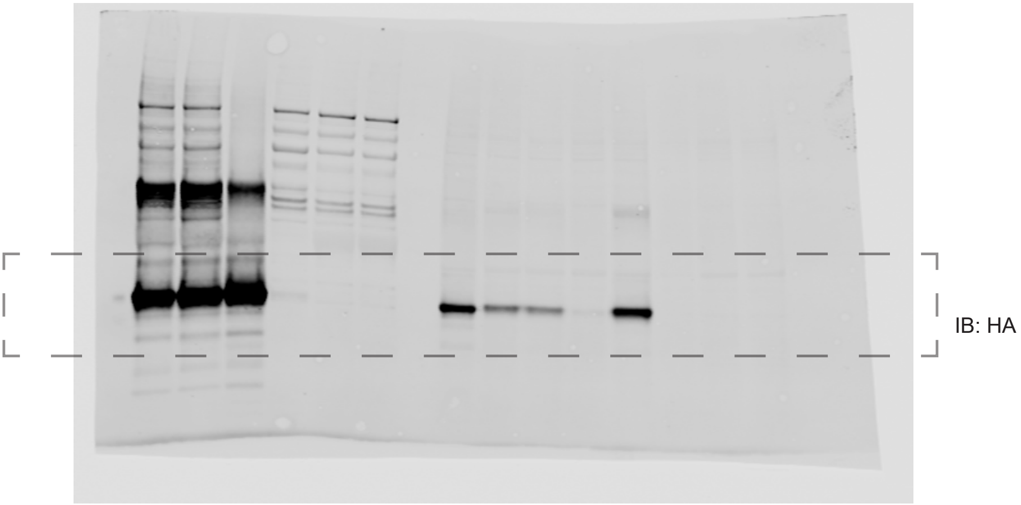

Supplemental Figure 1C, IP

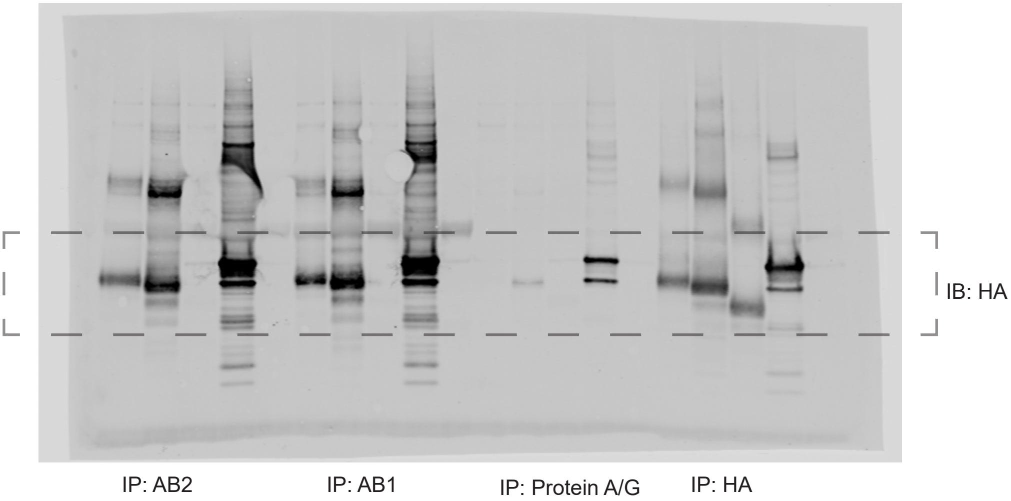

Supplemental Figure 1C, WCL

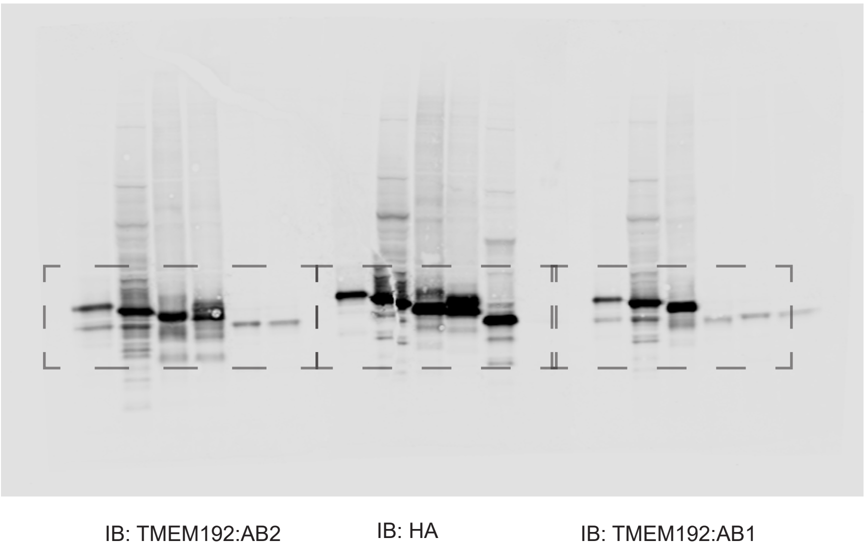

Supplemental Figure 4A

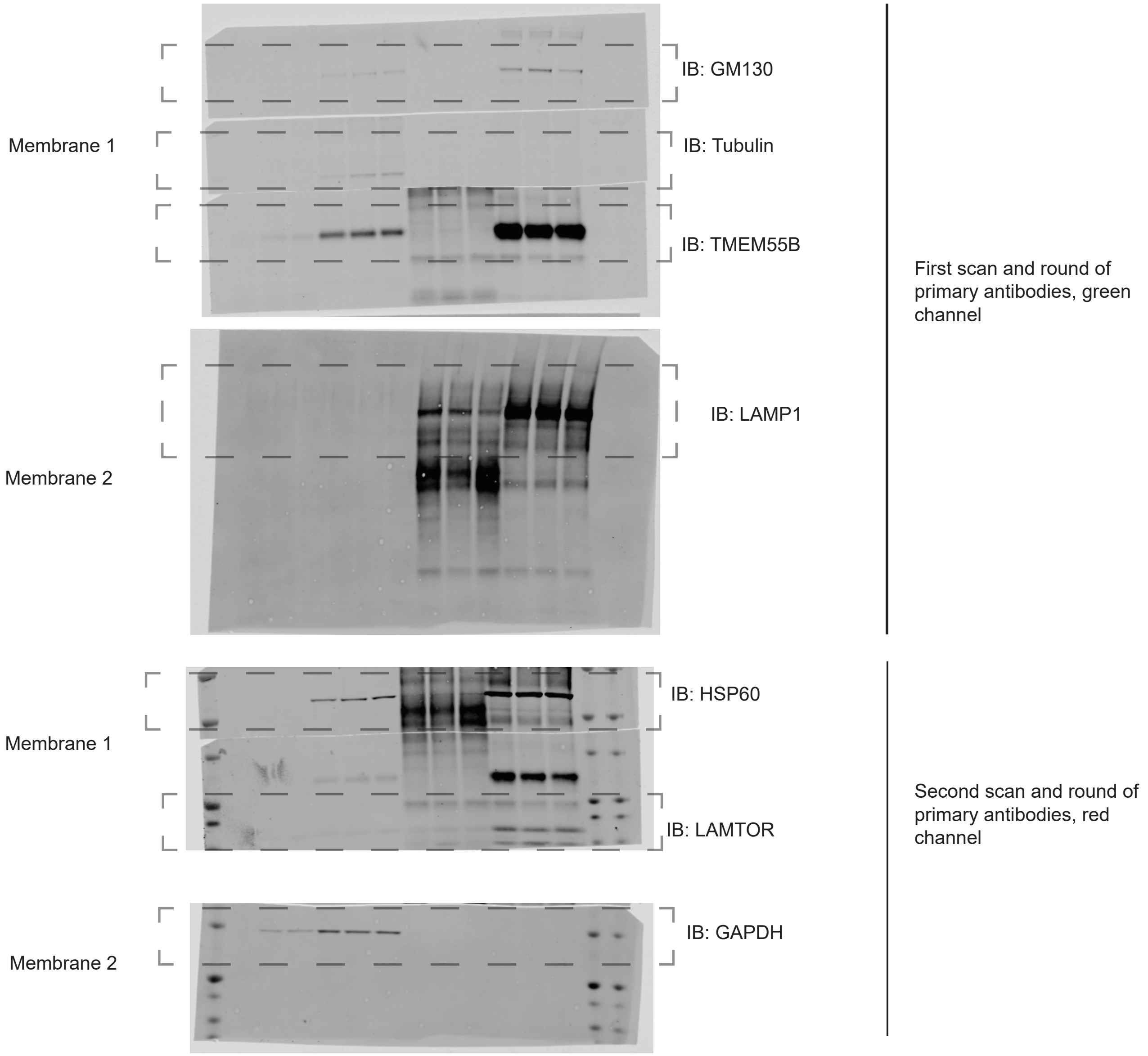

Supplement: Unedited blot and gel images [file jci-135-183592-s132.pdf]
